# Insights into the Acid–Base Properties of Pt^IV^–Diazidodiam(m)inedihyroxido Complexes from Multinuclear NMR Spectroscopy

**DOI:** 10.1002/chem.201002792

**Published:** 2011-09-16

**Authors:** Luca Ronconi, Ana M Pizarro, Ruth J McQuitty, Peter J Sadler

**Affiliations:** [a] School of Chemistry, University of EdinburghWest Mains Road, Edinburgh EH9 3JJ (UK); [b] Department of Chemistry, University of WarwickGibbet Hill Road, Coventry CV4 7AL (UK), Fax: (+44) 24-76523819 E-mail: P.J.Sadler@warwick.ac.uk

**Keywords:** acid–base properties, anticancer complexes, bioinorganic chemistry, NMR spectroscopy, p*K*_a_ values, platinum

## Abstract

Platinum(IV) am(m)ine complexes are of interest as potential anticancer pro-drugs, but there are few reports of their acid–base properties. We have studied the acid–base properties of three photoactivatable anticancer platinum(IV)-diazidodiam(m)ine complexes (*cis*,*trans*,*cis*-[Pt^IV^(N_3_)_2_(OH)_2_(NH_3_)_2_], *trans*,*trans*,*trans*-[Pt^IV^(N_3_)_2_(OH)_2_(NH_3_)_2_], and *cis*,*trans*-[Pt^IV^(N_3_)_2_(OH)_2_(en)]) using multinuclear NMR methods and potentiometry. In particular, the combination of both direct and indirect techniques for the detection of ^15^N signals has allowed changes of the chemical shifts to be followed over the pH range 1–11; complementary ^14^N NMR studies have been also carried out. A distinct p*K*_a_ value of approximately 3.4 was determined for all the investigated complexes, involving protonation/deprotonation reactions of one of the axial hydroxido groups, whereas a second pH-dependent change for the three complexes at approximately pH 7.5 appears not to be associated with a loss of an am(m)ine or hydroxido proton from the complex. Our findings are discussed in comparison with the limited data available in the literature on related complexes.

## Introduction

The platinum(II) complexes cisplatin, carboplatin, and oxaliplatin are currently approved drugs for the treatment of cancer. However, in spite of therapeutic success in the treatment of several types of tumors, their effectiveness is severely hindered by adverse side effects such as nausea, ototoxicity, neurotoxicity, myelosuppression, and nephrotoxicity. A second major drawback is tumor resistance, either acquired during cycles of therapy (as occurs in patients with, for instance, ovarian cancer) or intrinsic resistance (such as in patients with prostate, lung, or breast cancer).^1^ Thus, effort in designing new platinum drugs is aimed at making platinum-based therapy safer to patients, in particular by reducing or removing severe side effects, providing oral administration, and overcoming both intrinsic and acquired resistance.

In this regard, there has long been interest in cytotoxic six-coordinate platinum(IV) analogues, especially since they are often effective against tumors resistant to cisplatin. Platinum(IV) is a classically-inert low-spin d^6^ metal ion. Platinum(IV)-based anticancer drugs might be less toxic and more readily tolerated by normal cells. Increasing evidence suggests that for activity they require in vivo reduction to the corresponding platinum(II) species by biological reductants (e.g. ascorbic acid, intracellular glutathione). Such chemical reduction is highly dependent on the levels of reducing agents present in body fluids and cells, which can be variable. Platinum(IV) complexes may be regarded as inactive pro-drugs, and the coordinative binding of the resulting active platinum(II) species to DNA is widely considered to be responsible for their therapeutic activity.^2^ Several platinum(IV) drugs, including tetraplatin ([Pt^IV^Cl_4_(1,2-diaminohexane)]), satraplatin (*cis*,*trans*,*cis*-[Pt^IV^Cl_2_(acetate)_2_(NH_3_)(cyclohexylamine)]), and iproplatin (*cis*,*trans*,*cis*-[Pt^IV^Cl_2_(OH)_2_(isopropylamine)_2_]), have reached clinical trials and some positive responses have been reported in the early phases.^3^

Our current interest is in platinum(IV)-diazidodiam(m)inedihydroxido complexes, pro-drugs which are relatively inert toward reducing agents under physiological conditions. These non-toxic precursors might be selectively activated by light at the tumor site leading to the corresponding platinum(II) species, thereby potentially improving targeting and minimizing side effects.^4^, ^5^ Their behavior upon irradiation has been recently investigated by both experimental^6^–^8^ and theoretical^9^ studies, providing insights into the photoreaction pathways that are involved. The photoreaction pathways and types of photoproducts depend on the pH of the solution and other reaction conditions.

It is often assumed that neutral platinum(IV) ammine derivatives remain as neutral complexes in aqueous solution although am(m)ines coordinated to highly charged metals ions such as platinum(IV), or the isoelectronic gold(III), can be acidic with p*K*_a_ values close to 7, that is, within the physiological pH range.^10^ The presence of an anionic -NH_2_^−^ ligand as opposed to a neutral -NH_3_ ligand, for example, might be expected to have a major effect on cell uptake and distribution of platinum(IV) drugs, including binding to negatively charged DNA, and a detailed knowledge of the acid–base properties of platinum(IV) drugs is therefore important. Despite this, there appear to have been only a few reported studies.

To understand better the solution behavior of these platinum(IV)-diazidodiam(m)inedihydroxido pro-drugs and, consequently, the implications for their possible mechanism of action, we have made a detailed study of the acid–base properties of *cis*,*trans*,*cis*-[Pt^IV^(N_3_)_2_(OH)_2_(NH_3_)_2_] (**1**), *trans*,*trans*,*trans*-[Pt^IV^(N_3_)_2_(OH)_2_(NH_3_)_2_] (**2**), and *cis*,*trans*-[Pt^IV^(N_3_)_2_(OH)_2_(en)] (en=ethylenediamine) (**3**) using multinuclear NMR methods (combined with ^15^N-labeling of the complexes) and potentiometry. We discuss our findings in comparison with the limited literature data on related platinum(IV) complexes.

## Results

**NMR studies**: The acid–base properties of complexes **1**–**3** were investigated by 1D ^1^H, ^15^N, and ^14^N NMR spectroscopy. For the ^15^N NMR spectroscopy, ^15^N-labeling of the am(m)ine ligands was essential. The corresponding ^15^N-labeled am(m)ine analogues are referred to as ^**15**^**N-1**, ^**15**^**N-2**, and ^**15**^**N-3**.1
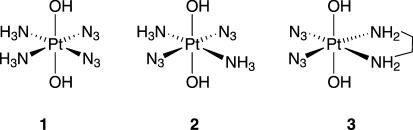


**Figure 1:**
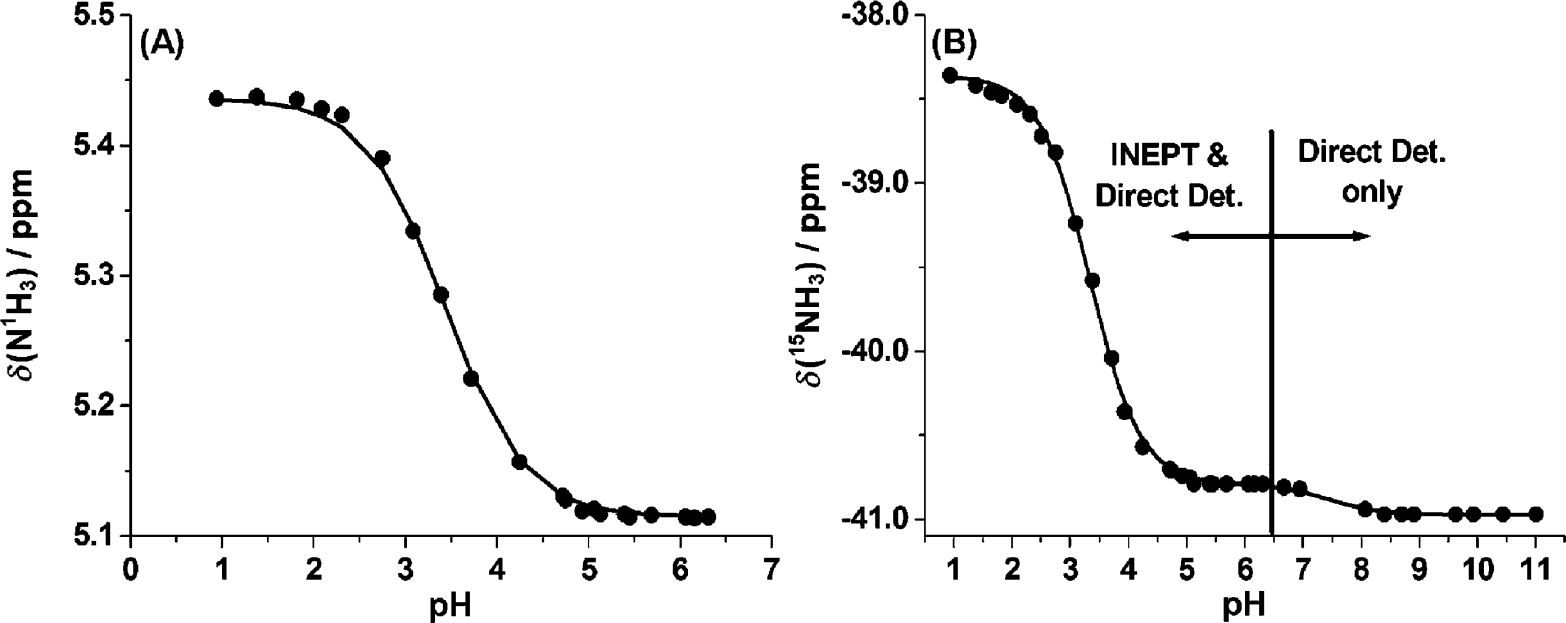
pH dependence of NMR chemical shifts for Pt^IV^–^15^NH_3_ of complex ^**15**^**N-1**. A) ^1^H (χ^2^=0.00002; *R*^2^=0.99908; p*K*_a1_=3.44±0.01), and B) ^15^N (χ^2^=0.00082; *R*^2^=0.99920; p*K*_a2_=3.34±0.01; p*K*_a2_=7.44±0.23).

The pH dependence of the ^1^H NMR chemical shifts of the ammine protons of complex ^**15**^**N-1** over the pH range 1.0–6.5 is shown in Figure 1 A. No proton signal was detected above pH 6.5 for coordinated ^15^NH_3_ due to the rapid exchange of ammine protons with the solvent. By fitting the experimental data to Equation (1) (see the Experimental Section), a value of p*K*_a1_=3.44±0.01 was determined.

1D ^15^N{^1^H} INEPT (Insensitive Nuclei Enhanced by Polarization Transfer) NMR experiments were also carried out over the same pH range but, again, no ^15^N peak was observable for ammine ligands above pH 6.5. On the other hand, acquisition of a succession of 1D ^15^N{^1^H} NMR spectra enabled the direct detection of the ^15^N-ammine signals over the entire range, from pH 1 to 11. Combined data for the pH dependence of the ^15^N chemical shifts of ammine ligands for complex ^**15**^**N-1** (Figure 1 B) were fitted to Equation (2) (see the Experimental Section), giving now two p*K*_a_ values p*K*_a1_=3.34±0.01 (in good agreement with that derived above from ^1^H NMR data) and p*K*_a2_=7.44±0.23, respectively. The latter is associated with only a small ^15^N shift change (ca. 0.1 ppm) and was not apparent from the ^1^H NMR data.

**Figure 2:**
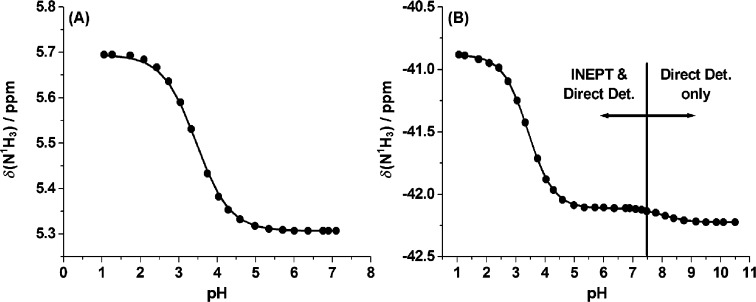
pH dependence of NMR chemical shifts for Pt^IV^–^15^NH_3_ of complex ^**15**^**N-2**. A) ^1^H (χ^2^=0.00001; *R*^2^=0.99963; p*K*_a1_=3.46±0.01), and B) ^15^N (χ^2^=0.00003; *R*^2^=0.99988; p*K*_a1_=3.42±0.01; p*K*_a2_=8.06±0.04).

Similar NMR experiments were carried out for complex ^**15**^**N-2** (Figure 2). The ^1^H data gave rise to p*K*_a1_ 3.46±0.01. As a consequence of the fast exchange of ammine protons, no 1D ^15^N{^1^H} INEPT NMR signals were detected above pH 7.5. Again, ^15^N signals of the ammine ligands were recorded over the pH range 1–11 by direct detection 1D ^15^N{^1^H} NMR experiments. The overall ^15^N NMR data for complex ^**15**^**N-2** (Figure 2 B) gave rise to p*K*_a1_=3.42±0.01, and an apparent p*K*_a2_=8.06±0.04, although again this pH-induced change in the chemical shift was observable only by a small change in ^15^N chemical shift and not for ^1^H shifts.

**Figure 3:**
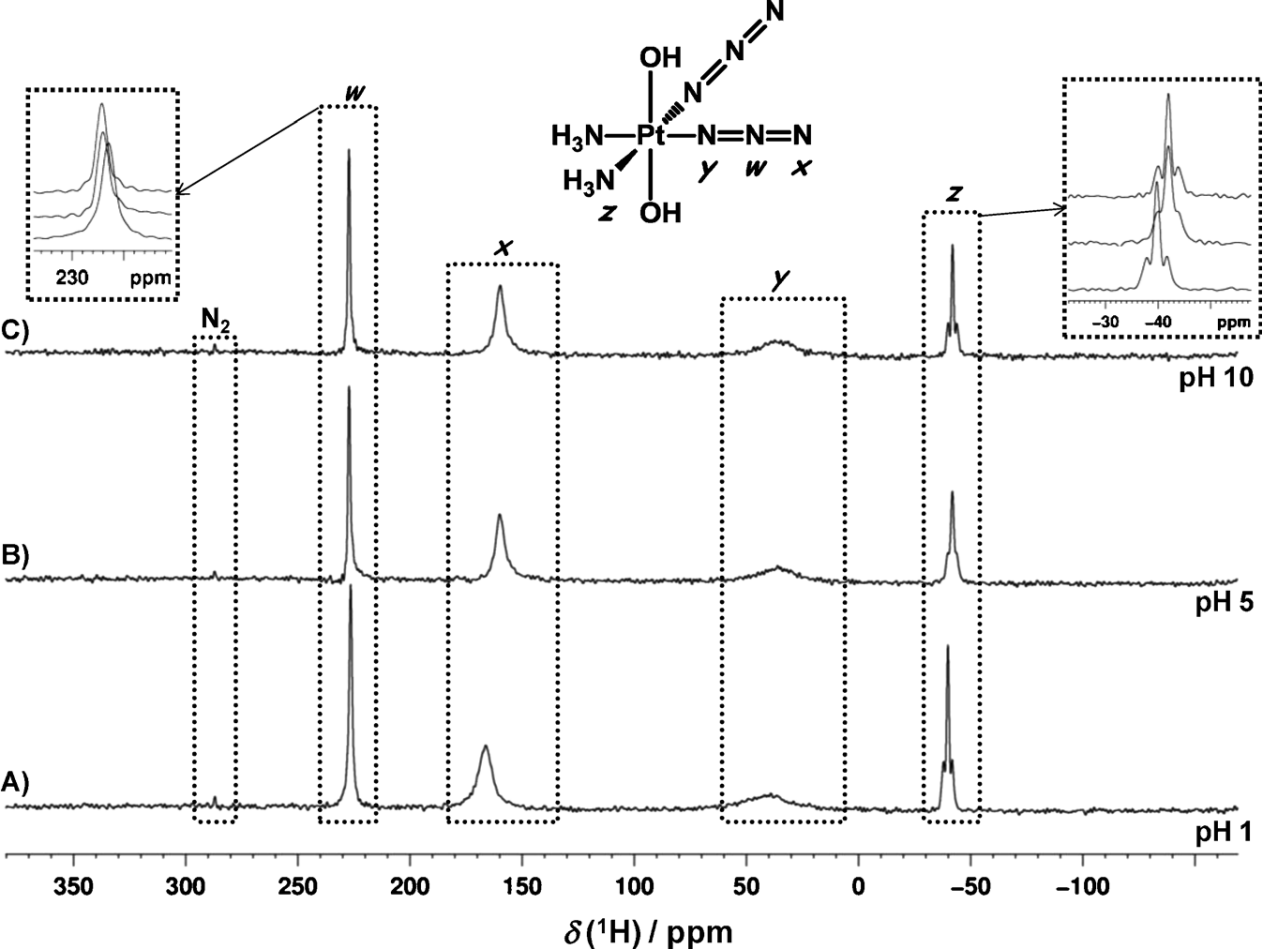
1D ^14^N{^1^H} NMR spectra of complex **1** in D_2_O in the dark at 298 K at A) pH 1, B) pH 5, and C) pH 10. Assignments (internal reference N_2_ at 287.5 ppm): coordinated azido ligand as labeled in structure (*w*=Pt^IV^–N*N*N, *x*=Pt^IV^–NN*N*, *y*=Pt^IV^–*N*NN); ammine ligand (*z*=Pt^IV^-*N*H_3_). Inserts of signals *w* and *z* show their dependence on the pH.

To confirm the observations by direct detection 1D ^15^N{^1^H} NMR experiments, 1D ^14^N{^1^H} NMR spectra were recorded at pH 1, 5 and 10 for complex **1** (Figure 3). ^14^N is high in natural abundance (99.6 %) but quadrupolar (*I*=1), and therefore resonances tend to be relatively broad, especially for the coordinated nitrogen of azide. Four ^14^N resonances were detected at approximately *δ*=230, 160, 35, and −40 ppm (relative to NH_4_Cl through N_2_ at *δ*=287.5 ppm as internal reference). In line with ^15^N experiments, the chemical shifts at pH 1 and 5 differ by several ppm (up to 6.32 for Pt^IV^–NN*N*, the terminal azido nitrogen, peak *x*), whereas little change was observed between pH 5 and 10. The Pt^IV^-*N*H_3_^14^N resonance shifts by 2.15 ppm from pH 1 to pH 5, but by only 0.06 ppm from pH 5 to 10 (upfield), similar to the shift changes for ^15^N resonances (2.23 and 0.10 ppm, respectively; see Table S1 in the Supporting Information). Analogous shift changes were observed in the 1D ^14^N{^1^H} NMR spectra of complex **2** recorded under similar experimental conditions (see Figure S1 in the Supporting Information).

**Figure 4:**
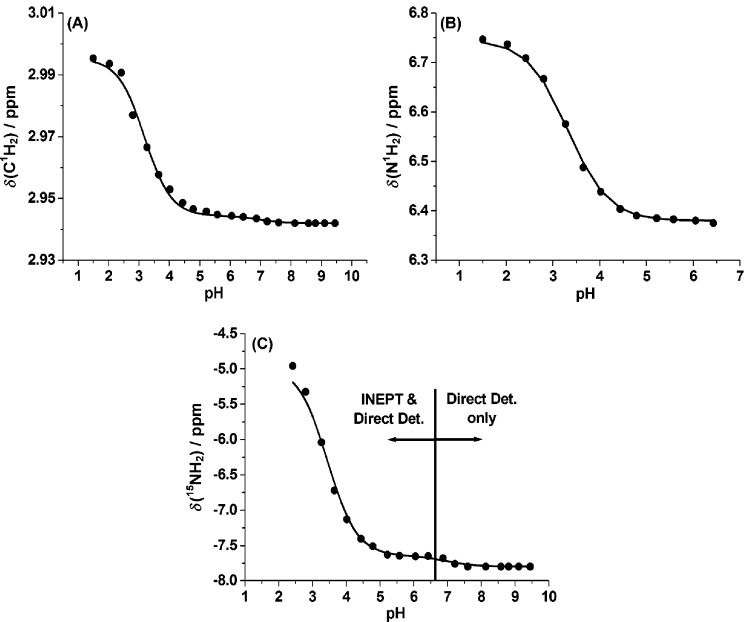
pH dependence of NMR chemical shifts of complex ^**15**^**N-3**. A) ^1^H chemical shift of -CH_2_- (χ^2^=0.00002; *R*^2^=0.99509; p*K*_a1_=3.38±0.03; p*K*_a2_=6.99±0.61), B) ^1^H chemical shift of Pt^IV^–^15^NH_2_- (χ^2^=0.00002; *R*^2^=0.99899; p*K*_a1_=3.32±0.01), and C) ^15^N chemical shift of Pt^IV^–^15^NH_2_- (χ^2^=0.00522; *R*^2^=0.99951; p*K*a_1_=3.45±0.31; p*K*_a2_=7.00±0.50).

To evaluate the effect of different amine ligands, the acid–base properties of the ethylenediamine (en) derivative *cis*,*trans*-[Pt^IV^(N_3_)_2_(OH)_2_(^15^N_2_-en)] (^**15**^**N-3**) were also investigated. The ^1^H NMR chemical shift changes for the ethylenediamine -CH_2_- groups over the pH range 1.5–10 (Figure 4 A) were fitted to Equation (2), giving p*K*_a1_=3.38±0.03 and p*K*_a2_=6.99±0.61. The large error in p*K*_a2_ is due to the extremely small upfield shift of the -CH_2_- peak for this step. The dependence of ^1^H (Figure 4 B) and ^15^N (Figure 4 C) NMR chemical shifts of the Pt^IV^–^15^NH_2_- group on pH were also determined. Direct 1D ^15^N{^1^H} NMR detection was successfully used over the entire pH range, whereas 1D ^1^H and ^15^N{^1^H} INEPT techniques were used only below pH 6.5, due to the fast exchange of the amine protons. From combined ^15^N data (Figure 4 C) a p*K*_a1_ value of 3.45±0.31 was obtained, in good agreement with the value of 3.32±0.01 obtained by fitting ^1^H NMR amine proton chemical shifts (Figure 4 B). From ^15^N data, a p*K*_a2_ of 7.00±0.50 (Figure 4 C), was also determined.

**HPLC studies**: RP-HPLC was used to determine the purity of compounds **1**–**3** using a near neutral (pH 6) aqueous mobile phase. Compounds **1** and **3** each gave rise to a single major peak under the experimental conditions (see Figure S2 in the Supporting Information). For complex **2**, the major peak with a retention time of approximately 1.6 min was accompanied by a minor peak at approximately 1.7 min, which accounted for about 16 % of the total peak area. The minor peak (see Figure S2A in the Supporting Information) seemed to increase in concentration over time and it was not observed when the chromatographic separation started less than 5 min after the compound was dissolved (see Figure S2B in the Supporting Information). Despite working under conditions of minimal light, this species can be attributed to a photoproduct, possibly the *cis*-isomer,^11^ due to the high photosensitivity of this compound.

Additional peaks were observed for complexes **1** and **3** at retention times 5.2 and 4.7 min, respectively, when a more acidic mobile phase was used (0.1 % TFA water/0.1 % TFA acetonitrile), using the same mobile phase gradient (data not shown). These impurities account for 0.4 % for compound **1**, and 0.3 % for compound **3**, and were therefore not characterized. However, they can be tentatively assigned to photoreduction products.^12^

**Figure 5:**
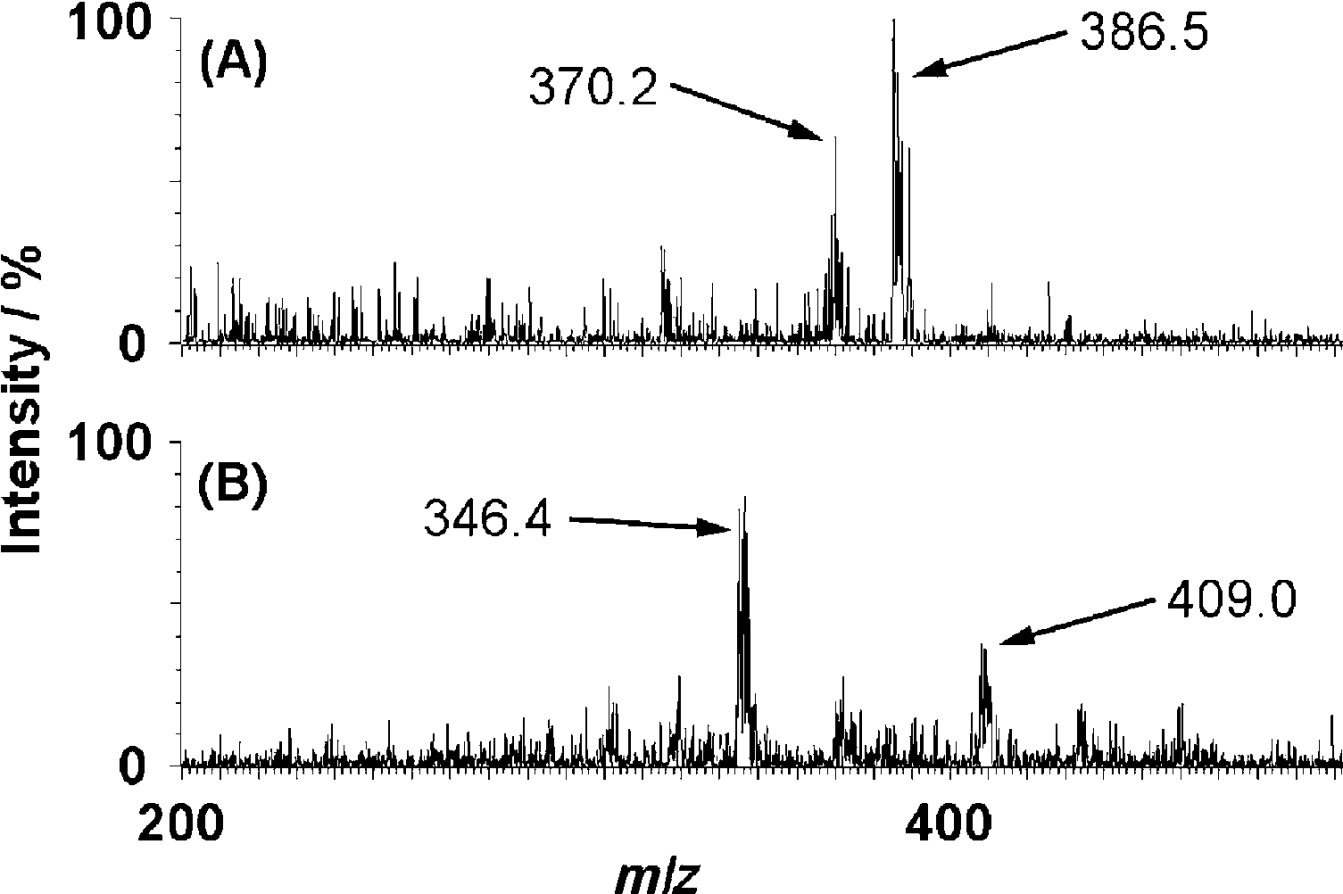
ESI mass spectra of aqueous solutions containing 0.8–1 mg mL^−1^ of complex **1** in A) positive-ion mode at pH 4.9 and B) negative-ion mode at pH 9.3.

**ESI-MS studies**: The purity of the investigated platinum(IV)-diazidodiam(m)inedihydroxido complexes was also studied by ESI-MS. As an example, the mass spectra for compound **1** under acidic conditions (pH 4.9) in positive-ion mode and under basic aqueous solution (pH 9.3) in negative-ion mode are shown in Figure 5 A and Figure 5 B, respectively.

The major ESI-MS peak for the observed positive ions at low cone voltage (+15 V) appears at *m/z* 386.5, consistent with the species [**1**+K]^+^ (calcd *m/z* 386.3). An additional peak of lower intensity was detected at *m/z* 370.2 assignable to the species [**1**+Na]^+^ (calcd *m/z* 370.2). ESI-MS spectra obtained in negative-ion mode (cone voltage: −15 V) in basic solution (pH 9.3) show a peak at *m/z* 346.4, consistent with the species [**1**−H]^−^ (calcd *m/z* 346.2). Similar experiments were performed for the *trans*-isomer **2** under the same experimental conditions. Again, the major ESI-MS peaks for the observed positive ions in acidic aqueous solution (pH 4.7) are consistent with [**2**+Na]^+^ and [**2**+K]^+^, whereas negative ion peaks can be assigned to [**2**−H]^−^ (data not shown), similar to the results obtained for the *cis*-isomer **1**.

**Potentiometric titrations**: The p*K*_a_ values for complex **1** were determined by potentiometric pH titrations in aqueous solution, and only one p*K*_a_ value at 3.56±0.03 could be determined. No dissociation of protons from the complex at pH values close to 7 was observed.

## Discussion

NMR titration methods are widely used to determine proton dissociation constants (*K*_a_). Proton dissociation from an ionizable group usually results in shifts of the NMR peaks from nearby nuclei in a molecule. Such shifts occur when the rate of proton exchange is ‘fast’ on the NMR time scale and represents the weighted average of the chemical shifts of the protonated and deprotonated forms. pH titration curves are then analyzed by curve-fitting approaches (e.g. Henderson–Hasselbalch equation-based algorithms), allowing p*K*_a_ determinations. A major advantage of this method over other techniques is that the results are not greatly affected by the presence of minor impurities and the shift changes often allow the protonation site to be identified unambiguously.^13^

NMR titrations are based on the evaluation of the changes of chemical shifts of *I*=1/2 nuclei such as ^1^H, ^31^P, and, usually upon isotopic enrichment, for example with ^13^C or ^15^N. In this regard, ^15^N NMR spectroscopy has been commonly employed in the study of platinum-based anticancer drugs as ^15^N-labeled derivatives can often be prepared from readily available starting materials. ^15^N NMR chemical shifts are sensitive to the nature of the *trans* ligand in platinum-am(m)ine derivatives and provide a powerful method for identifying the ligands in the coordination spheres of both platinum(II) and platinum(IV) complexes.^17^ In particular, 2D [^1^H,^15^N] HSQC (Heteronuclear Single Quantum Correlation) NMR spectroscopy has been exploited to study hydrolysis profiles and p*K*_a_ values of bound aqua ligands in aqua adducts of platinum am(m)ine compounds.^14^–^17^ However, there are only a few reports in the literature concerning the acid–base properties of non-aqua ligands bound to platinum and other metal complexes (e.g. am(m)ine protons).^10^, ^18^, ^19^ Coordination of the nitrogen atom of am(m)ines to a highly charged metal ion can result in a dramatic increase in the acidity of am(m)ine protons so that their p*K*_a_ values can be determined in aqueous solution.^20^

Direct detection is the most common technique for the observation of 1D ^15^N NMR spectra of all types of nitrogen atoms. Unfortunately, ^15^N suffers from low natural abundance (0.365 %), although this can be overcome by ^15^N-enrichment, a low and negative magnetogyric ratio (*γ*=−2.712×10^7^ rad T^−1^ s^−1^), low relative sensitivity (0.022 relative to ^13^C), and often also from long relaxation times.^21^ The indirect INEPT method improves the sensitivity of NMR experiments with low-abundance and low magnetogyric ratio nuclei. Specifically, the net effect is the non-selective polarization transfer from protons to ^15^N nuclei with an appropriate ^1^H-^15^N coupling. This polarization transfer method enhances signal intensity by transferring the greater population differences of high-γ spins (^1^H) onto their scalar spin-coupled low-γ partners (^15^N), leading to the amplification of the heteronucleus signal, increased sensitivity and shorter accumulation times.^22^

On the basis of these considerations, we assumed that when a ^15^N NMR signal is detected by both 1D ^15^N{^1^H} INEPT and direct detection 1D ^15^N{^1^H} NMR techniques the exchange of the ammine protons (interpreted in terms of base-catalyzed hydrogen exchange of coordinated am(m)ines in aqueous solution) is ‘slow’ on the NMR time scale. On the contrary, when it is recordable only through direct-detection 1D ^15^N{^1^H} NMR experiments, proton exchange is likely to be more rapid.

The use of both direct and indirect detection of ^15^N NMR parameters allowed detection of two distinct pH-dependent structural changes for all three platinum(IV) diazido complexes studied here. The first, with an associated p*K*_a1_ of approximately 3.4, can be assigned to the deprotonation of one of the axial hydroxide groups. By fitting the ^15^N chemical shifts of Pt^IV^–^15^N-am(m)ine at different pH to Equation (2) (see the Experimental Section) p*K*_a1_ values of 3.34±0.01 (Figure 1 B), 3.42±0.01 (Figure 2 B), and 3.45±0.31 (Figure 4 C) have been determined for ^**15**^**N-1**, ^**15**^**N-2**, and ^**15**^**N-3**, respectively (in good agreement with the values 3.44±0.01 (Figure 1 A), 3.46±0.01 (Figure 2 A) and 3.38±0.03/3.32±0.01 (Figure 4 A and Figure 4 B) obtained by fitting the related ^1^H NMR data to Equation (1) (see the Experimental Section).

**Figure 6:**
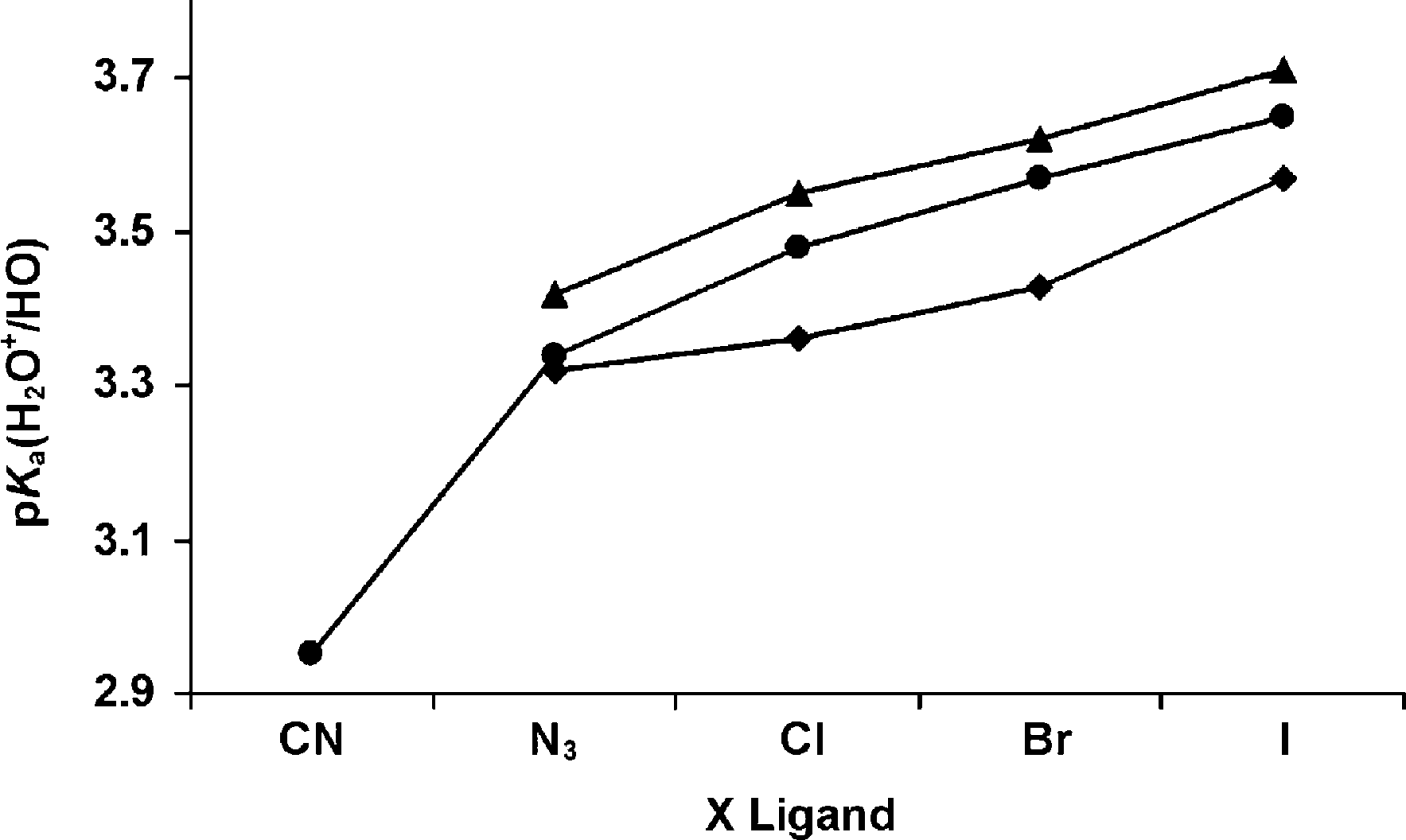
p*K*_a_ values for an axial aqua ligand in the platinum(IV) diam(m)ine complexes: (•) *cis*,*trans*,*cis*-[Pt^IV^(X)_2_(OH)(OH_2_)(NH_3_)_2_]^+^ (X=Cl, Br, I, CN, N_3_), (▴) *trans*,*trans*,*trans*-[Pt^IV^(X)_2_(OH)(OH_2_)(NH_3_)_2_]^+^ (X=Cl, Br, I, N_3_), and (♦) *cis*,*trans*-[Pt^IV^(X)_2_(OH)(OH_2_)(en)]^+^ (X=Cl, Br, I, N_3_).^23^–^26^

For all the complexes, p*K*_a1_ values associated with the protonation/deprotonation reactions of one of the axial hydroxido groups are very similar. Remarkably, the replacement of the two *cis*-ammine ligands with a chelating ethylenediamine has little effect on the acidity of the axial aqua/hydroxido groups. The p*K*_a1_ values calculated for complexes **1**–**3** are consistent with the few data available in the literature.^23^–^26^ Reported Pt–OH_2_/Pt–OH p*K*_a_ values, obtained from potentiometric measurements, for platinum(IV) diam(m)ineaqua derivatives of the type *cis*,*trans*,*cis*-[Pt^IV^(X)_2_(OH)(OH_2_)(NH_3_)_2_]^+^ (X=Cl, Br, I, CN), *trans*,*trans*,*trans*-[Pt^IV^(X)_2_(OH)(OH_2_)(NH_3_)_2_]^+^ (X=Cl, Br, I), and *cis*,*trans*-[Pt^IV^(X)_2_(OH)(OH_2_)(en)]^+^ (X=Cl, Br, I) are shown in Figure 6, and compared with the values obtained here for complexes **1**–**3**. It is notable that these values show a trend in accordance with the electron-withdrawing power of the X ligands: CN> N_3_> Cl> Br> I. Thus, the greater the electron-withdrawing power of the equatorial ligands, the lower the Pt^IV^–OH_2_/Pt^IV^–OH p*K*_a_ value.

The second pH-dependent event, occurring in the pH range 7–8, requires a separate discussion and interpretation. For the three complexes ^**15**^**N-1**, ^**15**^**N-2**, and ^**15**^**N-3**, ‘p*K*_a2_’ values of 7.44±0.23 (Figure 1 B), 8.06±0.04 (Figure 2 B) and 7.00±0.50 (Figure 4 C) have been determined. At first glance, these values might be associated with acid–base reactions involving the equatorial am(m)ine ligands. However, if a pH-induced deprotonation of a coordinated am(m)ine were to occur, a larger shift of the ^15^N NMR resonance of the am(m)ine ligand would be expected, as well as the ^1^H NMR signals of the -CH_2_- ethylenediamine in complex **3**. Again, contrary to the hypothesis of am(m)ine deprotonation is the fact that the ‘p*K*_a2_’ determined for compound **2** is about 0.6 units greater than that of its *cis*-isomer **1**, whereas the higher *trans* influence of the azido ligand compared to ammonia,^27^ would be expected to give rise to a higher p*K*_a2_ for an ammine *trans* to an azido ligand.^18^

^14^N NMR has proved useful for the study of platinum(IV) diazido complexes,^6^–^8^ especially if pulse sequences are used which minimize acoustic ringing so that very broad peaks can be observed. In this regard, results obtained by direct detection 1D ^15^N{^1^H} NMR studies have been confirmed by 1D ^14^N{^1^H} NMR experiments carried out for compounds **1** and **2** at pH 1, 5 and 9/10 (Figure 3 and Figure S1 in the Supporting Information); changes of the ^14^N chemical shifts for the ammine ligands are consistent with those observed in ^15^N spectra recorded at the same pH values (see Tables S1 and S2 in the Supporting Information). In addition to the small chemical shift changes observed for the ammine nitrogens between pH 5 and 10, the greater shifting of the ^14^N signals of the azido ligands supports the idea of a pH-dependent change over that pH range.

Only a few p*K*_a_ values for coordinated am(m)ines have been reported in the literature (Table 1), and none of them refers to complexes similar to those investigated here. Comparing the available data with those for compounds **1**–**3**, only complexes of the type [Pt^IV^(Am)_*n*_]^4+^ (Am=NH_3_ (*n*=6) or en (*n*=3)) show lower or, at least, comparable p*K*_a_ values to those associated with the second inflexion point. However, this is attributed to the fact that these complexes carry a high positive charge. Compared to the neutral platinum(IV) am(m)ine derivatives in Table 1, the ‘p*K*_a2_’ value for the am(m)ine protons in the diazido complexes should be dramatically more basic. For example, if we compare complex **2** with complex *trans*-[Pt^IV^(CN)_4_(NH_3_)_2_], due to the lower electron-withdrawing properties of the azido and hydroxido ligands in comparison with the cyanido ligand, it would be predicted that the p*K*_a_ of the ammine ligands in **2** is greater than 12.1, and not consistent with a value close to 7.5.

**Table 1:** p*K*_a_ values reported for coordinated am(m)ines in mixed-ligand platinum(IV) am(m)ine complexes in aqueous solution at 298 K.

Platinum(IV) complexes	p*K*_a_
*trans*-[Pt^IV^(CN)_4_(NH_3_)_2_]	12.1^10^
*trans*-[Pt^IV^Cl(CN)_3_(NH_3_)_2_]	12.7^10^
*trans*-[Pt^IV^Br(CN)_3_(NH_3_)_2_]	12.8^10^
*trans*-[Pt^IV^I(CN)_3_(NH_3_)_2_]	13.0^10^
*mer*-[Pt^IV^Cl_3_(NH_3_)_3_]^+^	11.1^10^, ^18^
*trans*-[Pt^IV^Cl_2_(NH_3_)_4_]^2+^	11.3^10^, ^18^, ^28^
*cis*-[Pt^IV^Cl_2_(NH_3_)_4_]^2+^	9.5^10^, ^18^, ^28^
*trans*-[Pt^IV^Cl_2_(en)_2_]^2+^	10.3^18^
*trans*-[Pt^IV^Br_2_(en)_2_]^2+^	9.3^18^
*trans*-[Pt^IV^I_2_(en)_2_]^2+^	8.0^18^
*trans*,*cis*-[Pt^IV^Cl_2_(NH_3_)_2_(en)]^2+^	10.2[18] ^[a]^
*trans*,*cis*-[Pt^IV^Br_2_(NH_3_)_2_(en)]^2+^	9.9[18] ^[a]^
*trans*,*cis*-[Pt^IV^I_2_(NH_3_)_2_(en)]^2+^	6.8[18] ^[a]^
[Pt^IV^(OH)(NH_3_)_5_]^3+^	9.5[18, 24, 29] ^[b]^
[Pt^IV^Cl(NH_3_)_5_]^3+^	8.4[10, 18, 25, 28] ^[b]^
[Pt^IV^Br(NH_3_)_5_]^3+^	8.3[10, 18, 25] ^[b]^
[Pt^IV^Cl(NH_3_)_3_(en)]^3+^	7.7/8.9[30] ^[c]^
[Pt^IV^(NH_3_)_6_]^4+^	7.7^10^, ^18^, ^28^
[Pt^IV^(en)_3_]^4+^	5.5^18^, ^28^

[a] Not specified whether ammine or ethylenediamine protons are involved. [b] Not specified whether *cis* or *trans* (to either hydroxide or halide) ammine ligands are involved. [c] Values refer to two unspecified isomers.

Taken together, these observations suggest that the ‘p*K*_a2_’ values of approximately 7.5 calculated for complexes **1**–**3** at the second inflexion points are unlikely to be associated with the deprotonation of an equatorial am(m)ine ligand. This is consistent with the potentiometric titration for compound **1**. From this, only one p*K*_a_ value of 3.56±0.03 could be determined, assignable to the protonation/deprotonation of an axial hydroxido group, in good agreement with the p*K*_a1_ values determined by NMR spectroscopy (3.34/3.44). No other acid–base equilibrium was detected potentiometrically, in particular within the range 7–8.

The lack of detection of proton release for this step in combination with NMR data, suggests that the observed NMR effects are due to a pH-dependent proton movement but not release form the complex, for example, an am(m)ine proton adopting an H-bonding position with an axial hydroxido perhaps mediated by a water molecule or hydroxide ion. Hambley and co-workers^31^ have recently determined p*K*_a_ values for axial hydroxido ligands in platinum(IV) complexes using cyclic voltammetry and obtained values that lie in the same range as those reported here. Moreover, they have suggested that intermolecular bridging mechanisms are likely to be involved in both reduction and ligand exchange, which seem to be influenced by protonation of the hydroxido ligands. In this regard, the possible formation of bridged complexes upon deprotonation of the am(m)ine ligands cannot be ruled out, including amido-bridged species, although the limited relevant data available in the literature do not aid interpretation of our experimental results.^32^, ^33^

The possibility can be considered that such pH-dependent behavior near pH 7 is due to deprotonation of a minor species in solution which interacts rapidly on the NMR timescale with the investigated complexes. In this respect, it has been reported that association of trace platinum(II) impurities (which might arise for example, from the starting platinum(II) precursors, or from photoreduction of the platinum(IV) starting materials when briefly exposed to light during sample preparation) with the corresponding platinum(IV) complexes can increase the substitution rates of ligands coordinated to the platinum(IV) center.^34^ Since HPLC studies have shown that the complexes used in this NMR study have a high state of purity (>99 %, see Figure S2 in the Supporting Information), it seems reasonable to suppose therefore that the NMR data can be interpreted as arising from one predominant species in solution.

## Conclusion

In this work, we have investigated the acid–base properties of three platinum(IV) diazidodiam(m)inedihydroxido complexes, *cis*,*trans*,*cis*-[Pt^IV^(N_3_)_2_(OH)_2_(NH_3_)_2_] (**1**), *trans*,*trans*,- *trans*-[Pt^IV^(N_3_)_2_(OH)_2_(NH_3_)_2_] (**2**), *cis*,*trans*-[Pt^IV^(N_3_)_2_(OH)_2_(en)] (**3**), by means of multinuclear NMR methods and potentiometry. These complexes belong to a class of photoactivatable platinum(IV) anticancer pro-drugs.^4^–^9^ In this regard, it is of particular interest to investigate whether the acid–base behavior of these platinum(IV) derivatives can influence their chemical and biological properties. Platinum(IV) complexes are relatively inert, so they are not expected to undergo facile ligand substitution reactions, but are likely to reach cellular target sites intact. To exert their antitumor activity, it is normally thought that platinum(IV) complexes must be first reduced by biological reductants to the corresponding more reactive platinum(II) species, so any pH influence is generally taken into account only with respect to their reduction pathways.^35^

pH titrations followed by NMR spectroscopy allowed us to determine two distinct pH-dependent events with mid-points at approximately pH 3.4 and pH 7.5; this was made possible by the combination of both direct and indirect techniques for the detection of ^15^N and ^14^N chemical shifts. By comparison with the few data reported in the literature, it is reasonable to conclude that the lower p*K*_a_ value involves protonation/deprotonation of one of the axial hydroxido groups. In contrast, the second pH-dependent phenomenon cannot be reasonably be attributed to the deprotonation of one of the equatorial am(m)ine ligands, since a larger shift of the ^15^N and ^14^N NMR resonances upon deprotonation of the coordinated am(m)ine would be expected. In addition, no proton release was detected potentiometrically at around pH 7.5. Also there are no reported p*K*_a_ values lower than 10 for neutral platinum(IV)-am(m)ine complexes. Only complexes with overall charges of 2+, 3+, and 4+ have p*K*_a_ values below 8 (Table 1).

It seems likely therefore that the second inflexion point in the titration curve at appropximately pH 7–8 is associated with an intramolecular structural change in which a proton changes its environment in the complex but does not dissociate. The nature of the structural change will require further investigation. Perhaps it could involve for example a movement of the axial OH proton (or an NH proton) towards a terminal azido nitrogen—effectively pH-induced deprotonation resulting in an increase in basicity of bound azide. Such a structural change could influence the photoreduction pathways for these complexes and their biological activity.

## Experimental Section

**Materials**: K_2_[Pt^II^Cl_4_] was purchased from Precious Metals Online; KI, NaN_3_, KOH, NaCl, NaOH, KClO_4_, and HCl from Fisher Scientific; AgNO_3_, NH_4_Cl, ^15^NH_4_Cl, ethylenediamine dihydrochloride, ^15^N_2_-ethylenediamine dihydrochloride, 1,4-dioxane, and D_2_O from Sigma–Aldrich; H_2_O_2_ (30 %) from Prolabo; CH_3_CN and HClO_4_ from Fisons.

**Syntheses**: Complexes *cis*,*trans*,*cis*-[Pt^IV^(N_3_)_2_(OH)_2_(NH_3_)_2_] (**1**), *trans*,*trans*,*trans*-[Pt^IV^(N_3_)_2_(OH)_2_(NH_3_)_2_] (**2**) and *cis*,*trans*-[Pt^IV^(N_3_)_2_(OH)_2_(en)] (**3**) were synthesized according to literature procedures.^36^, ^37^ ^15^NH_4_Cl and ^15^N_2_-ethylenediamine dihydrochloride were used as the source of ^15^N for the synthesis of the corresponding ^15^N-labeled analogues (^**15**^**N-1**, ^**15**^**N-2**, ^**15**^**N-3**).

**1**. Yield: 78–80 %; 1D ^1^H NMR (500.1 MHz, 90 % H_2_O/10 % D_2_O, pH 5.4, TSP): *δ*=5.12 ppm (Pt^IV^–^15^N*H*_3_, 6 H, ^1^*J*(^15^N–H)=73 Hz, ^2^*J*(^195^Pt–H)=46 Hz); 2D [^1^H, ^15^N] HSQC NMR (500.1/50.7 MHz, 90 % H_2_O/10 % D_2_O, pH 5.4, ^15^NH_4_Cl): *δ*=−40.8 ppm (Pt^IV^–^15^*N*H_3_, ^1^*J*(^195^Pt–^15^N)=261 Hz).

**2**. Yield: 72–73 %; 1D ^1^H NMR (500.1 MHz, 90 % H_2_O/10 % D_2_O, pH 5.4, TSP): *δ*=5.31 ppm (Pt^IV^–^15^N*H*_3_, 6 H, ^1^*J*(^15^N–H)=73 Hz, ^2^*J*(^195^Pt–H)=48 Hz); 2D [^1^H, ^15^N] HSQC NMR (500.1/50.7 MHz, 90 % H_2_O/10 % D_2_O, pH 5.4, ^15^NH_4_Cl): *δ*=−42.1 ppm (Pt^IV^–^15^*N*H_3_, ^1^*J*(^195^Pt–^15^N)=282 Hz).

**3**. Yield: 88–90 %; 1D ^1^H NMR (500.1 MHz, 90 % H_2_O/10 % D_2_O, pH 5.5, TSP): *δ*=2.95 ppm (Pt^IV^–^15^NH_2_–C*H*_2_, 4 H, ^2^*J*(^15^N–H)=1.4 Hz, ^3^*J*(^195^Pt–H)=24 Hz); *δ*=6.39 ppm (Pt^IV^–^15^N*H*_2_, 4 H, ^1^*J*(^15^N–H)=76 Hz, ^2^*J*(^195^Pt–H)=44 Hz); 2D [^1^H, ^15^N] HSQC NMR (500.1/50.7 MHz, 90 % H_2_O/10 % D_2_O, pH 5.5, ^15^NH_4_Cl): *δ*=−7.7 ppm (Pt^IV^–^15^*N*H_2_, ^1^*J*(^195^Pt-^15^N)=277 Hz).

**Warning!** Heavy metal-azido complexes are known to be shock-sensitive detonators. We encountered no problems in this study, but these materials should be handled with extreme caution, especially not to put pressure on them in the crystalline form.

All experiments were carried out with minimal exposure to light due to the high photosensitivity of the complexes.

**NMR spectroscopy**: All NMR spectra were acquired in 90 % H_2_O/10 % D_2_O at 298 K on a Bruker DMX500 spectrometer using a TBI [^1^H, ^13^C,X] probe-head equipped with *z*-field gradients or a Bruker AV111-600 spectrometer. Data processing was carried out using MestReNova version 5.2.5 (Mestrelab Research S.L.) or with TOPSPIN version 2.1 (Bruker U.K. Ltd.).

Typical acquisition parameters for 1D ^1^H NMR spectra (^1^H: 500.13 MHz): 16 transients, spectral width 7.5 kHz, using 32k data points and a delay time of 2.0 s. Water suppression was achieved using a 55.0 dB power level presaturation. Data were processed by using exponential weighting with a resolution of 0.5 Hz and a line-broadening threshold of 0.1 Hz. ^1^H chemical shifts were referenced to TSP by using internal 1,4-dioxane at 3.764 ppm.

Typical acquisition parameters for 1D ^15^N{^1^H} INEPT and 1D ^15^N{^1^H} NMR spectra (^15^N: 50.70 MHz): 128 transients, spectral width 15 kHz, using 64k data points and a delay time of 4.0 s. Sequences were optimized for ^1^*J*(^15^N–H)=76 Hz, and ^1^H decoupling was achieved by using WALTZ16 pulse sequence. Data were processed by using exponential weighting with a resolution of 0.3 Hz and a line-broadening threshold of 6.0 Hz. ^15^N chemical shifts were referenced to external ^15^NH_4_Cl 1.5 m in 1 m HCl at 0.00 ppm.

Typical acquisition parameters for 2D [^1^H, ^15^N] HSQC NMR spectra (^1^H: 500.13/^15^N: 50.70 MHz): 512 transients of 16 scans/block, spectral width 7.5/4.6 kHz, using 2k/2k data points and a delay time of 1.5 s. Sequences were optimized for ^1^*J*(^15^N–H)=76 Hz, and ^1^H decoupling was achieved by using WALTZ16 pulse sequence. Data were processed using cosine-square weighting with a resolution of 2.2/3.7 Hz and a line-broadening threshold of 0.7/1.0 Hz. ^1^H chemical shifts were referenced to TSP by using internal 1,4-dioxane at 3.764 ppm. ^15^N chemical shifts were referenced to external 1.5 m ^15^NH_4_Cl in 1 m HCl at 0 ppm.

Typical acquisition parameters for 1D ^14^N{^1^H} NMR spectra (^14^N: 43.35 MHz): 32k transients, spectral width 24 kHz, 16k data points and a delay time of 0.20 s. Due to acoustic resonance, the anti-ringing proton-decoupled ARINDEC pulse sequence was used.^38^ Data were processed using an exponential line-broadening of 5 Hz and a resolution of 1.5 Hz. ^14^N chemical shifts were referenced to internal N_2_ at 287 ppm (relative to external 1.5 m NH_4_Cl in 1 m HCl).

**pH measurement**: pH values were measured at room temperature directly in the NMR tube with a Corning 145 pH-meter equipped with an Aldrich microcombination electrode, calibrated with standard Aldrich buffer solutions at pH 4, 7 and 10.

**pH titrations followed by NMR spectroscopy and p*K***_**a**_ **determination**: The NMR samples for pH titrations contained 15–20 mm platinum(IV) complex in 0.6 mL 90 % H_2_O/10 % D_2_O. The pH values of the solutions were adjusted by using dilute KOH or HClO_4_, and NMR spectra were recorded.

**Scheme 1:**
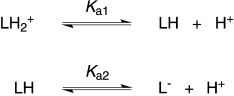
General scheme for a two-step acid–base equilibrium.

The experimental pH titration data were fitted to the formulae in Equations (1) and (2) derived from Henderson–Hasselbalch equations (for one or two p*K*_a_ values, respectively) according to the general acid–base equilibria in Scheme 1.


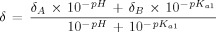
(1)


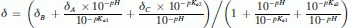
(2)

*K*_a1_ and *K*_a2_ are the dissociation constants for species LH_2_^+^ and LH, respectively, and *δ*_A_, *δ*_B_, and *δ*_C_ are the limiting chemical shifts of species LH_2_^+^, LH and l^−^, respectively. The pH titration curves (chemical shift vs. pH) were fitted using the program ORIGIN version 6.0 (Microcal Software Ltd.).

**Mass spectrometry (ESI-MS)**: Positive- and negative-ion electrospray mass spectra were obtained on a Micromass Platform II mass spectrometer. The samples were prepared in water to a final concentration of 0.8–1.0 mg mL^−1^, and pH was adjusted using dilute KOH or HClO_4_ solutions. Samples (100 μL) were injected directly into the source by a syringe pump at a flow rate of 0.5 mL h^−1^, and the ions were produced in an atmospheric pressure ionization (API) ESI ion source. The source temperature was 383 K, and the drying gas (N_2_) flow rate was 300 L h^−1^. A potential of 3.5 kV (capillary voltage) was applied to the probe tip (HV lens voltage: 0.5 kV; multiplier: 650 V), and the cone voltage was varied between (±) 5 and 25 V, depending on sensitivity. Mass spectra were recorded over the scan range 200–800 Da at a scan time rate of 2 s and a resolution of 2 *m/z*. Data acquisition and processing were carried out using MassLynx Software version 3.5 (Waters Corp.).

**Potentiometric titrations**: Potentiometric studies were carried out with a 721 NET Titrino autotitrator (Metrohm) equipped with a Metrohm combined pH glass electrode (Ag/AgCl) with 3 m KCl internal filling solution. The NaOH aqueous solution was standardized against potassium hydrogen phthalate with phenolphthalein as indicator. The electrode was calibrated with dilute standard acid and alkali solutions, thus defining pH=−log[H^+^] (p*K*_W_=14.28).^39^ The linearity of the electrode response and carbonate contamination of the standardized NaOH solution (0.0933 m) was determined by Gran’s method^40^ and was found to be less than 1 %. In a typical pH-metric determination, an aqueous solution (57 mL) of complex **1** (1.021 mm) treated with 4.45 equivalents of HClO_4_ was titrated with the standardized NaOH solution. The temperature of the solutions (298 K) in the covered water-jacketed cell was kept constant by a Julabo circulating bath. The ionic strength (*I*) was adjusted to 0.1 with KClO_4_. The protonation/deprotonation constants were calculated from the titration data by using the program HyperQuad 2000 version 2.1 (HyperQuad Solutions).^41^

**HPLC studies**: HPLC investigations of complexes **1–3**, were carried out on an Agilent 1100 system using a reverse-phase C18 column (150×4.6 mm, 5 μm particle size, Agilent Zorbax Eclipse Plus). Solvents were either H_2_O (solvent A) and CH_3_CN (solvent B), at a flow rate of 1.0 mL min^−1^ eluting with a gradient 5–80 % B over 60 min. All injections were 50 μL of aqueous solutions of approximately 20–30 μm of each platinum(IV) complex. All solutions were made up and handled with minimal exposure to light. The detection wavelength was 254 nm (close to the *λ*_max_ of the azide-to-Pt^IV^ charge-transfer bands of these complexes).

## Supplementary material

Detailed facts of importance to specialist readers are published as ”Supporting Information”. Such documents are peer-reviewed, but not copy-edited or typeset. They are made available as submitted by the authors.
