# Supplementary material for: Insights into the Acid–Base Properties of PtIV–Diazidodiam(m)inedihyroxido Complexes from Multinuclear NMR Spectroscopy
Source: Chemistry. 2011 Sep 16;17(43):12051–8. doi: 10.1002/chem.201002792 (PMC3743210; doi:10.1002/chem.201002792)
Supplement: Supplementary file 1 [file chem0017-12051-SD1.pdf]

CHEMISTRY
A EUROPEAN JOURNAL

Supporting Information

© Copyright Wiley-VCH Verlag GmbH & Co. KGaA, 69451 Weinheim, 2011

Insights into the Acid–Base Properties of Pt^{IV}–Diazidodiam(m)inedihyroxido Complexes from Multinuclear NMR Spectroscopy

Luca Ronconi,[a] Ana M. Pizarro,[b] Ruth J. McQuitty,[b] and Peter J. Sadler*[b]

chem_201002792_sm_miscellaneous_information.pdf

Table S1. pH-induced ^{14}N chemical shift changes for complex **1** as determined by 1D $^{14}N\{^1H\}$ NMR.

**Assignment**	**Δδ (pH 1 to pH 5)**	**Δδ (pH 5 to pH 10)**
w, Pt^{IV}–N***N***N	0.55	0.11
x, Pt^{IV}–NN***N***	6.32	0.28
y, Pt^{IV}–***N***NN	n.d.[a]	n.d.[a]
z, Pt^{IV}–***N***H_3	2.15 (2.23[b])	0.06 (0.10[b])

[a] Accurate determination of the peak shift for *y* was not possible due to the broadness of the signal. [b] Chemical shift change of the coordinated ammine as determined by $^{15}N\{^1H\}$ NMR.

Table S2. pH-induced ^{14}N chemical shift changes for complex **2** as determined by 1D $^{14}N\{^1H\}$ NMR.

**Assignment**	**Δδ (pH 1 to pH 5)**	**Δδ (pH 5 to pH 9)**
w, Pt^{IV}–N***N***N	1.00	0.05
x, Pt^{IV}–NN***N***	5.33	0.22
y, Pt^{IV}–***N***NN	n.d.[a]	n.d.[a]
z, Pt^{IV}–***N***H_3	0.93 (1.12[b])	0.07 (0.11[b])

[a] Accurate determination of the peak shift for *y* was not possible due to the broadness of the signal. [b] Chemical shift change of the coordinated ammine as determined by $^{15}N\{^1H\}$ NMR.

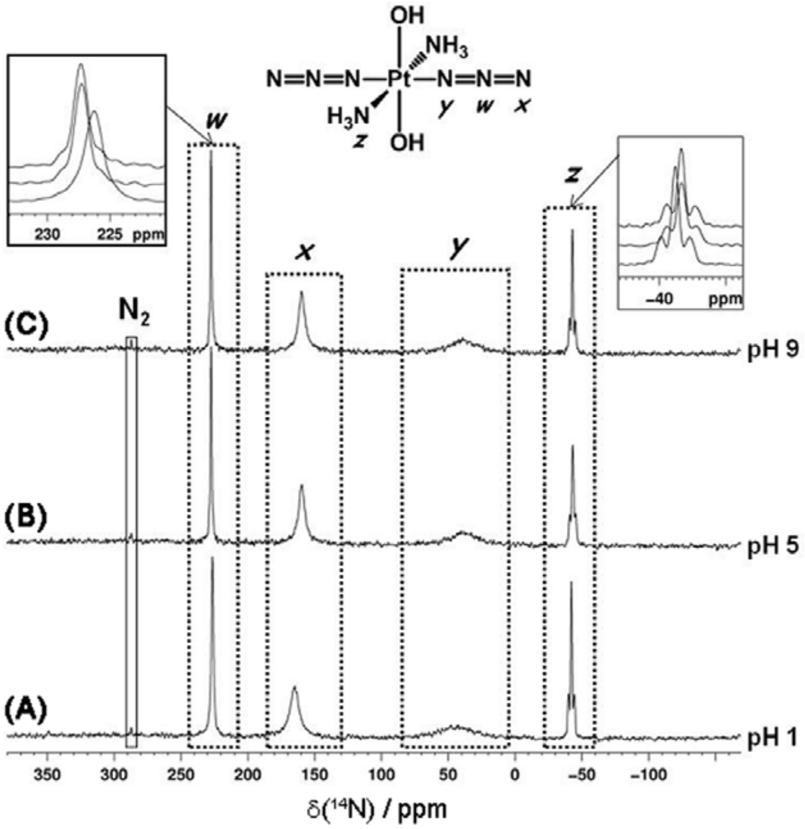

Figure S1. 1D $^{14}N\{^1H\}$ NMR spectra of complex **2** in D_2O in the dark at 298 K at (A) pH 1, (B) pH 5, and (C) pH 9. Assignments (internal reference N_2 at 287 ppm): coordinated azido ligand as labeled in structure (*w* = Pt^{IV}–N***N***N, *x* = Pt^{IV}–NN***N***, *y* = Pt^{IV}–***N***NN); ammine ligand (*z* = Pt^{IV}–***N***H_3). Inserts of signals *w* and *z* show their dependence on the pH.

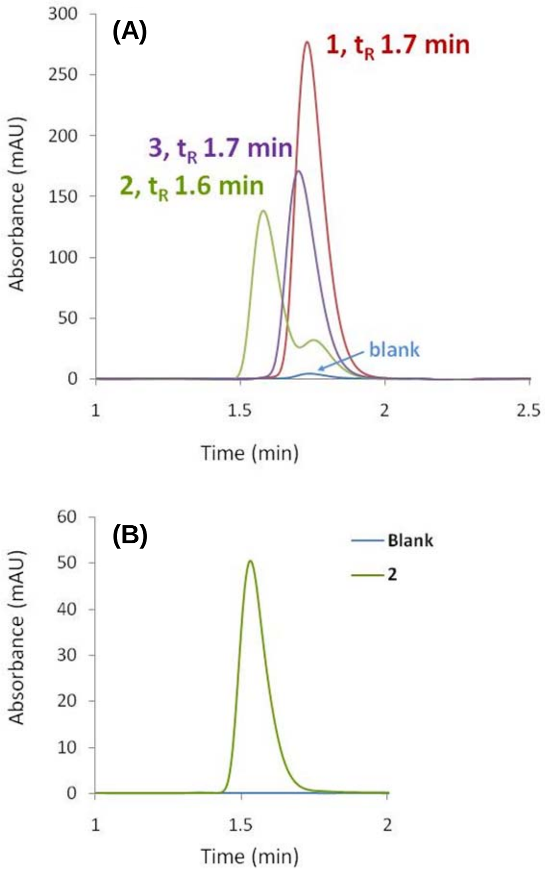

Figure S2. HPLC chromatogram in 5% water/95% acetonitrile (pH 6) with corresponding retention times at λ = 254 nm of (A) compounds **1-3** and blank for comparison, (B) compound **2** (< 5 min after the sample was dissolved) and blank for comparison.
